# Currarino Syndrome Presenting in Adulthood: A Rare Case

**DOI:** 10.7759/cureus.36497

**Published:** 2023-03-21

**Authors:** Abhijit Verma, Sanjeev Attry, Kulbhushan Haldenia, Vijay K Gupta, Puneet Bansal

**Affiliations:** 1 Department of Neurosurgery, NIMS University, Jaipur, IND; 2 Surgical Gastroenterology, NIMS University, Jaipur, IND

**Keywords:** chatgpt, anorectal anomaly, sacrococcygeal agenesis, anterior sacral meningocoele, currarino syndrome, currarino triad

## Abstract

Currarino syndrome or Currarino triad is a complex condition consisting of congenital anomalies. The triad consists of anterior sacral mass (meningocele, teratoma or dermoid/epidermoid cyst), sacral bone defect (hypoplasia, agenesis ), anorectal malformation/congenital anorectal stenosis. This condition is named after Dr Guido Currarino, an Italian-American paediatric radiologist, who first described it in 1975. This needs a multidisciplinary treatment approach. We describe a case of successfully managed young adult with Currarino syndrome. The latest artificial intelligence tool, Chat Generative Pre-Trained Transformer (ChatGPT), helped to write this case report.

## Introduction

Anterior sacral meningocele (ASM) is an anomaly where the meninges protrude into retroperitoneal and presacral space through a ventral sacral defect. Currarino syndrome is a complex condition variably comprised of characteristic congenital anomalies of the sacrum, anal and rectal defect with presacral soft tissue mass. It is a triad consisting of anterior sacral mass (meningocele, teratoma or dermoid/epidermoid cyst), sacral bone defect (hypoplasia, agenesis), anorectal malformation/congenital anorectal stenosis. This condition is named after Dr Guido Currarino, an Italian-American pediatric radiologist, who first described it in 1975, however, not all three features are always present. We report a 30-year-old adult with ASM presenting with constipation which improved after surgery.

## Case presentation

A 30-year-old male presented to our center with complaints of chronic constipation. Patient had noticed that multiple times this constipation was severe and he used to take laxatives and enema as per advice of local practitioner which helped him to pass stools. Since the last three years he started feeling weakness, tiredness, and fatigue for which he visited medical college in Jaipur where he was investigated and diagnosed with severe anemia and had multiple blood transfusion. There was no history associated with pain abdomen, vomiting, difficulty in passing urine, difficulty in walking.

The adult weighted 47 kilograms with paleness noted in lower palpebral conjunctiva, nail beds and palmar skin. On chest examination bilateral air entry was normal. Per abdomen examination revealed soft non distended abdomen with no other mass palpable except for fecolith. Bowel sounds were normal. Anal tone was found to be normal on digital rectal examination but the lumen was narrow and finger could not be negotiated, which precluded higher examination. Clinically spine was found to be normal. There was no neurological deficit.

Hematological investigations showed hemoglobin 2.8 gm/dl, total leucocyte count 6.73 thousand, platelets 366 thousand; liver function test and kidney function tests were normal.

**Figure 1:**
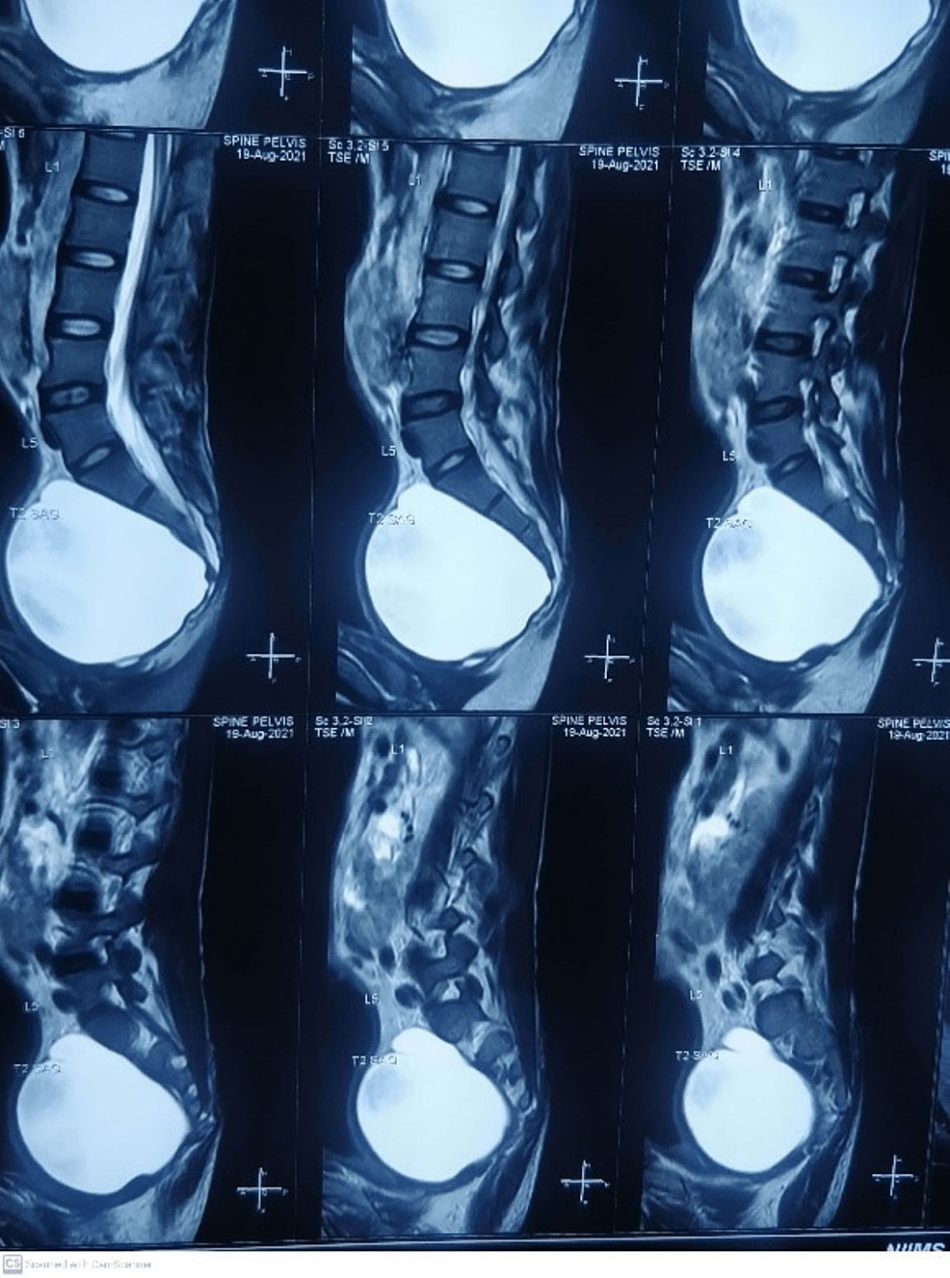
T2 Weighted MRI saggital cut showing large cystic mass anterior to sacrum

**Figure 2:**
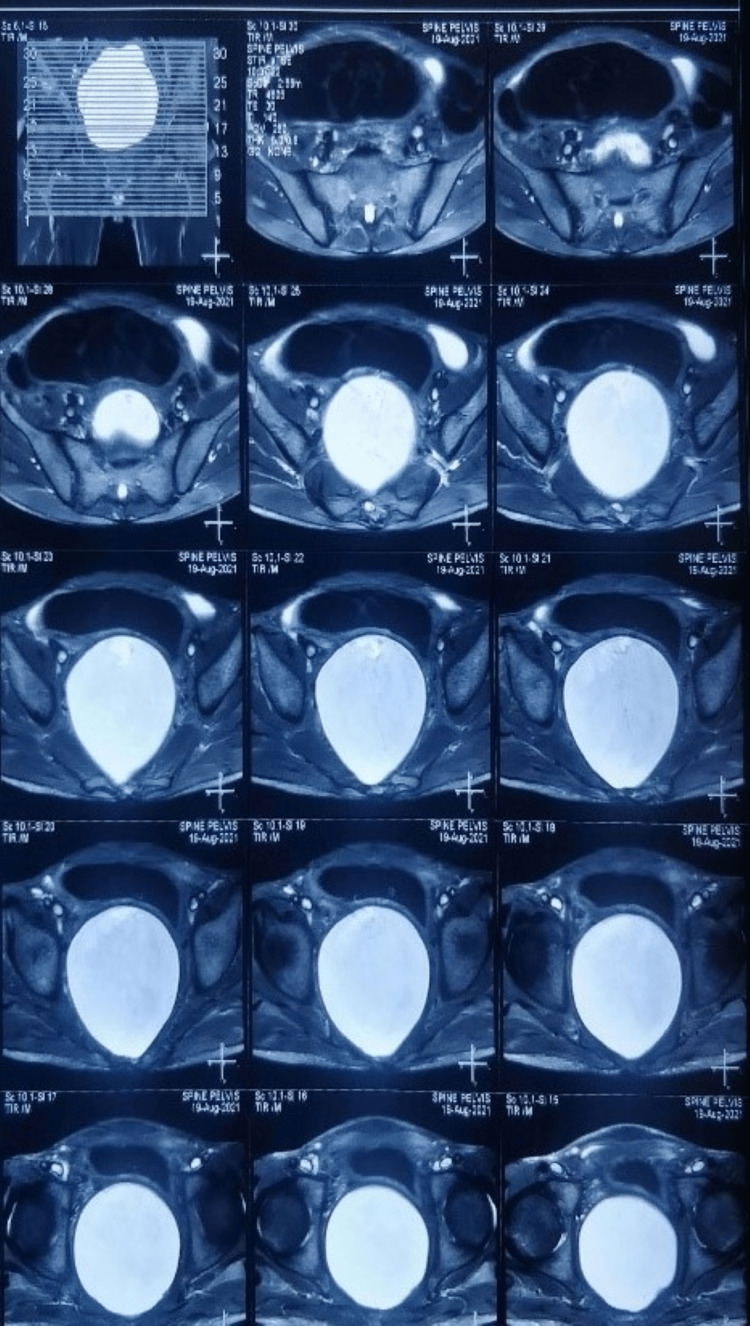
T2 Weighted axial cut showing large cystic mass anterior to sacrum

Ultrasound revealed right iliac fossa/left iliac fossa gaseous distended large bowel loops noted. MRI scan of pelvis revealed large heterogenous, non contrast enhancing mass anterior to upper lumbar spine with caliber of 25 × 7.5 centimeter, no diffusion restriction on diffusion weighted image and appearing to be anterior meningocele (Figures 1, 2).

**Figure 3:**
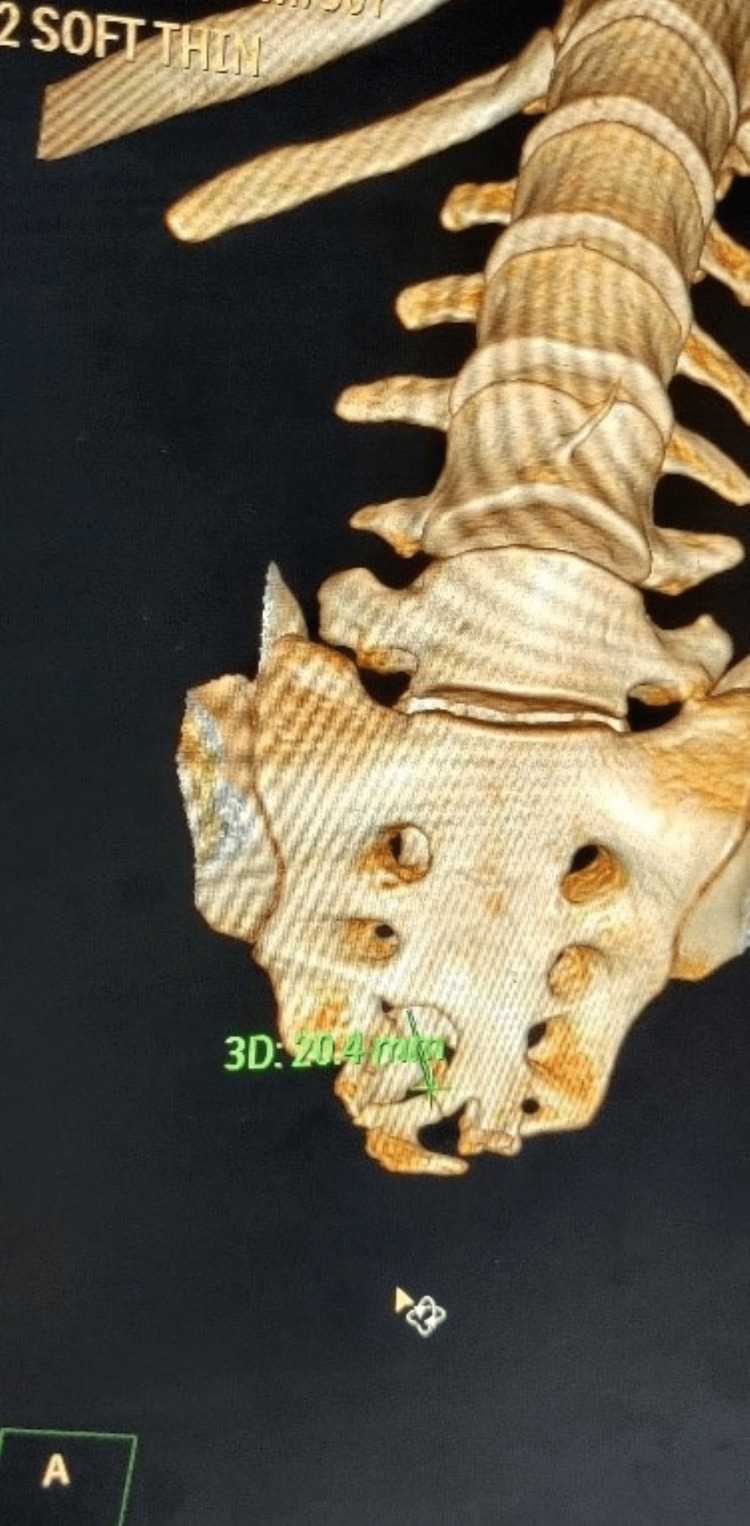
3D CT of sacrum showing defect in ventral part at S3 level

**Figure 4:**
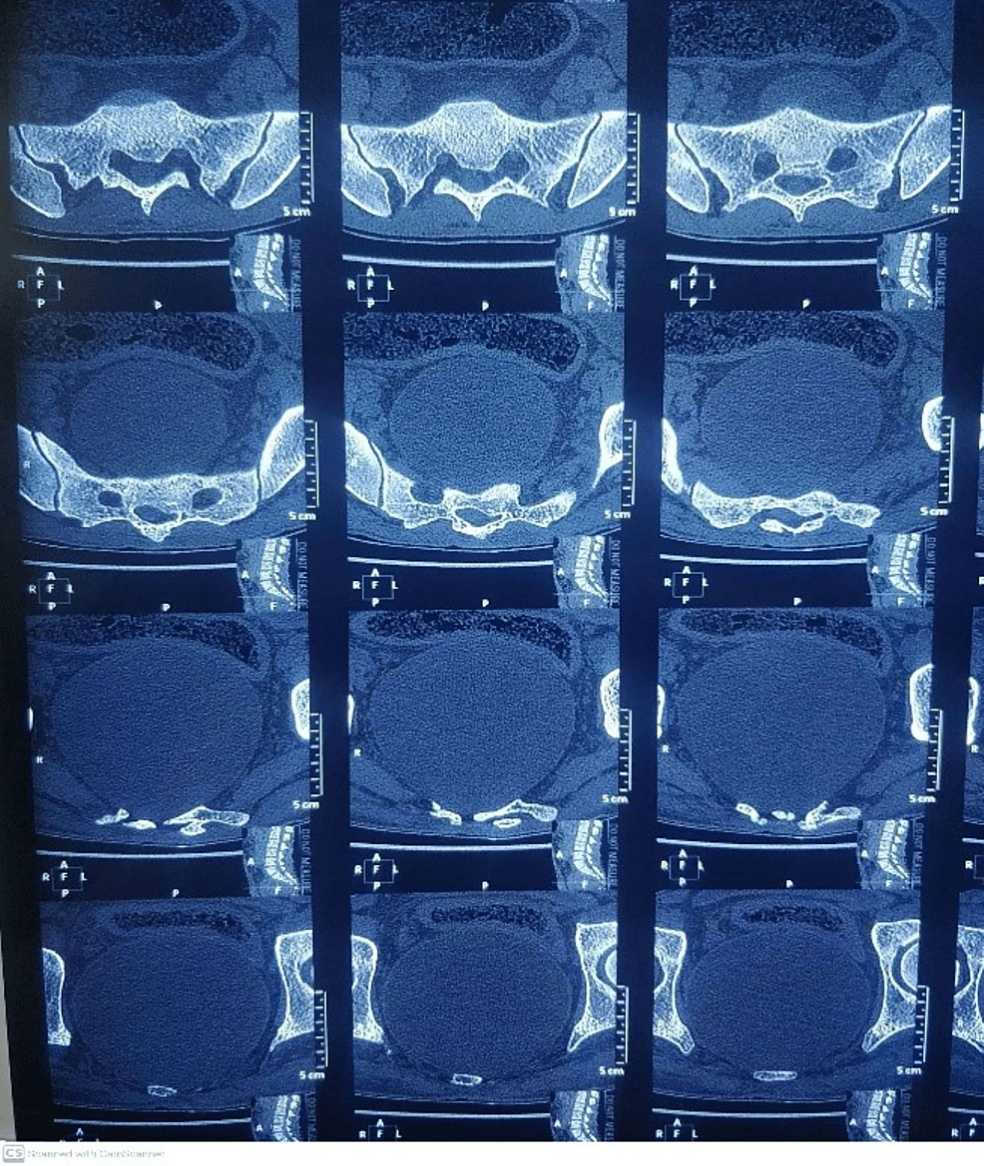
Non-contrast CT axial cut showing defect in ventral part of sacrum with a cystic mass anteriorly compressing rectum

Non-contrast computed tomography (NCCT) lumbo-sacral area revealed a large lobulated thick-walled cystic lesion measuring 10.5×9.9×10.7 centimeters seen in pelvis along sacral and coccyx vertebrae, bulging at S2-S3 (sacral vertebra 2 & 3) level, compressing and displacing surrounding bowel loops and soft tissue structures likely anterior meningocele along with ventral defect in S3-S4 vertebrae (Figures 3, 4). Echocardiogram did not show any associated cardiac anomaly. Sigmoidoscopy revealed stenosed and narrowed anal canal.

Patient was primed for planned surgery. Surgery was performed jointly by the Neurosurgery and Gastrosurgery teams.

Posterior midline approach was taken by the Neurosurgery team. Sacrum exposed and laminectomy done for S2,3,4 vertebrae. The outpouching of dura anteriorly through the sacral defect which was about 2cm×3cm in size was noted. No roots seen entering the cyst. The dural tube was seen terminating into the cyst. Meningocele cyst wall disconnected from the dural tube and marsupialization of cyst done. No evidence of any mass or collection other than cerebro spinal fluid (CSF)noted. Water tight closure of dural tube done at the terminal end. Muscle and skin closed in layers. This was followed by diversion loop sigmoid colostomy made in left iliac fossa and matured with Vicryl 3-0. There was no neurological deficit in the immediate postoperative period.

**Figure 5:**
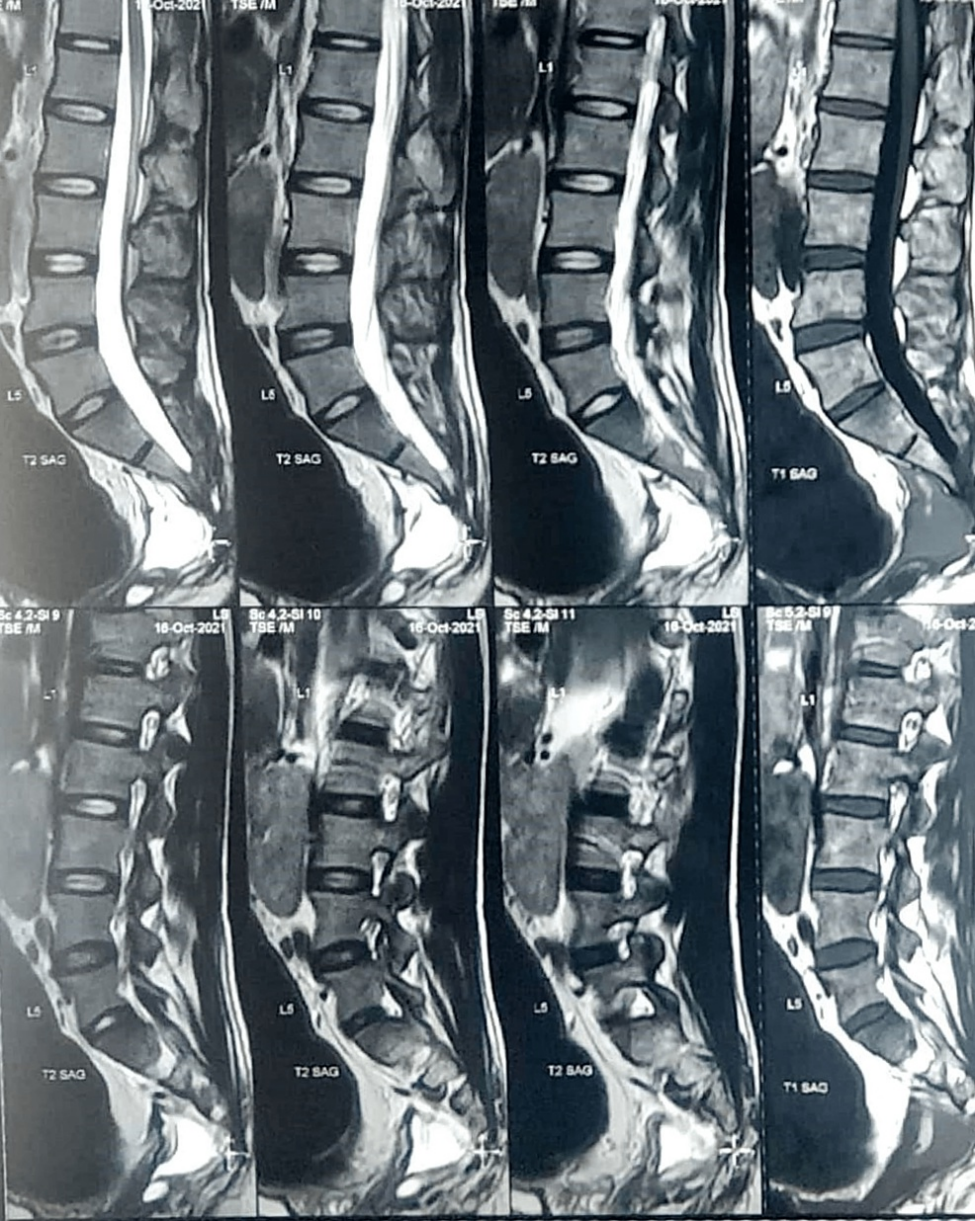
Postoperative three-month follow-up MRI T2 weighted sagittal cut showing regression of the meningocele

Postoperative course was uneventful and stoma was functioning at day two and patient was mobilized from day two postoperative. He was tolerating normal regular diet from postoperative day three. Wound was healthy and he was discharged at day five with advice to come to regular follow-up for anal dilatation. By doing regular anal dilatation, we achieved the target anal canal diameter in six weeks. After that stoma closure was done. Postoperative course was uneventful. He was able to pass stool through anal canal without any difficulty. He was in regular follow up with improving hemoglobin and gaining weight. Patient was neurologically intact. Postoperative follow-up MRI scan at three-month follow-up showed regression of the sacral meningocele (Figure 5).

## Discussion

ASM is a rare form of spinal dysraphism consisting of protrusion of dural sac anterior through a defect in the sacrum anteriorly [1-3]. ASM results from a fusion defect during sacral development. North et al. [4] have classified the possible mechanisms leading to ASM as: congenital (sacral bone defect, proliferation of arachnoid, connective tissue disorders; degenerative (ischemic lesion), traumatic (nerve root avulsion or hemorrhage); Iatrogenic (during surgery).

ASM is also a part of Currarino syndrome which includes anorectal malformations, sacral bony defect and presacral mass [5-7].

ASM can remain undiagnosed until adulthood due to its occult nature. Patients with this syndrome can have constipation, urological or neurological problems. ASM may lead to infertility and difficult labor in women and chronic constipation, urinary retention in young adults and children [8].

Secondary infection in the cyst can lead to pyocoele formation or meningitis [9]. Tethered cord could be associated with ASM leading to neurological signs [10]. Rarely, these can be occupied by epidermoid cyst [11] or could rupture into the rectum. ASM can be misdiagnosed as ovarian cyst [12].

‘Scimitar’ sign, a smooth curved unilateral sacral defect simulating shape of an Arabic sabre on plain X-ray, is considered to be pathognomonic of ASM [13]. Ultrasonography is the first investigation of choice as it can not only detect the cyst but can help to differentiate it from other pelvic cysts [14]. for further details of cyst, identifying boney defect and planning surgical intervention, CT and MRI scans are needed. It also helps to differentiate other pelvic masses (like teratoma) from ASM [15]. Endoscopy forms an important investigative tool to localize the stenosed part.

Most cases are managed surgically. Decompression of the cyst is the primary aim. It can be achieved by disconnecting meningocoele from the spinal subarchnoid space [16,17]. Dorsal approach done via laminectomy is preferred. Detethering of tethered cord is considered in the same sitting [18]. One has to be careful to preserve nerve roots in the vicinity to prevent postoperative neurological complications. 

In some cases, additional treatments may be necessary to manage symptoms and prevent complications. For example, physical therapy may be recommended to improve muscle strength and mobility, and medication may be prescribed to manage pain or bowel and bladder incontinence.

The latest artificial intelligence (AI) tool, Chat Generative Pre-trained Transformer (ChatGPT), was used to write the discussion. The format and information provided by ChatGPT were used after paraphrasing sentences to avoid plagiarism, as seen in the Appendix.

## Conclusions

It is important to note that the management of Currarino triad can be complex and may require long-term care and monitoring. Individuals with Currarino triad may experience permanent neurological problems and require ongoing care. With early diagnosis and appropriate treatment, most individuals are able to lead normal lives. However, it is always recommended to have regular follow-ups to ensure the best possible outcome.

## Figures and Tables

**Figure 6:**
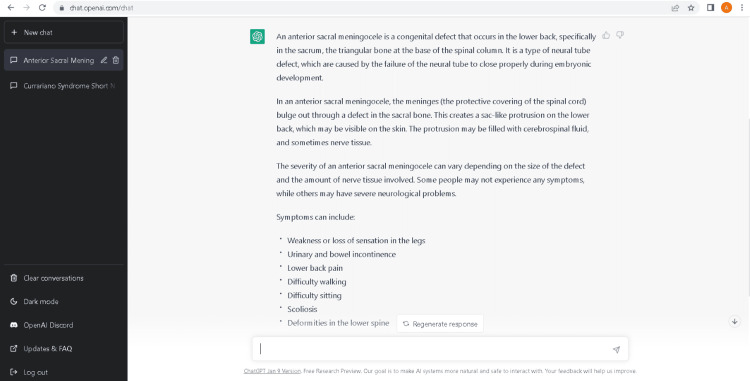
screen shot showing ChatGPT reply to my query

**Figure 7:**
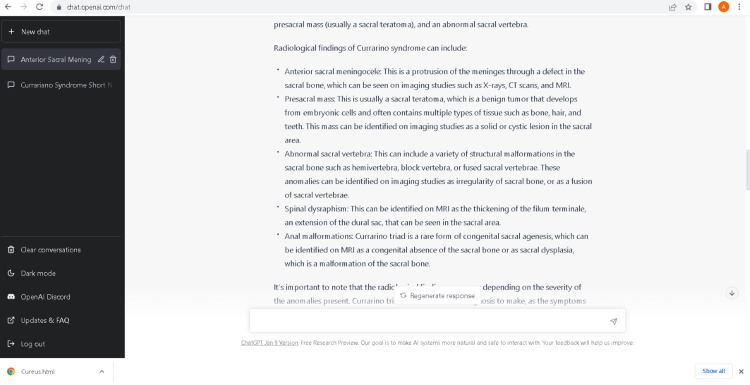
ChatGPT screen Shot

**Figure 8:**
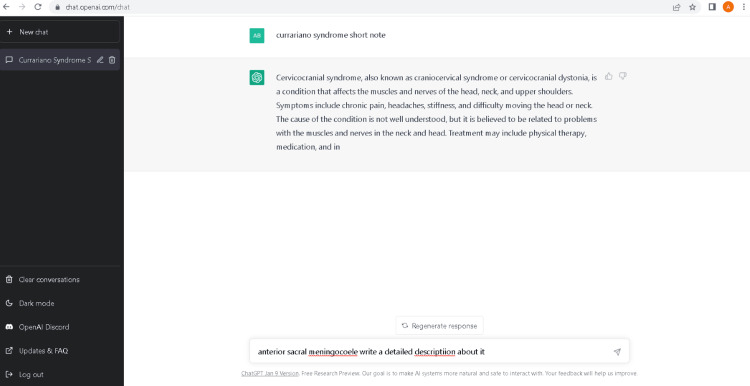
ChatGPT Screen Shot
